# A complex health services intervention to improve medical care in long-term care homes: study protocol of the controlled coordinated medical care (CoCare) study

**DOI:** 10.1186/s12913-019-4156-4

**Published:** 2019-05-24

**Authors:** Boris A. Brühmann, Christina Reese, Klaus Kaier, Margrit Ott, Christoph Maurer, Simone Kunert, Bruno R. Saurer, Erik Farin

**Affiliations:** 1 Institute of Medical Biometry and Statistics, Section of Health Care Research and Rehabilitation Research (SEVERA), Faculty of Medicine and Medical Center, University of Freiburg, Freiburg, Germany; 2 Institute of Medical Biometry and Statistics, Division Methods in Clinical Epidemiology, Faculty of Medicine and Medical Center, University of Freiburg, Freiburg, Germany; 3 Centre for Geriatric Medicine and Gerontology (ZGGF), Faculty of Medicine and Medical Center, University of Freiburg, Freiburg, Germany; 4 Association of Statutory Health Insurance Physicians Baden Wuerttemberg (KVBW), Stuttgart, Germany; 5 nubedian GmbH, Karlsruhe, Germany

**Keywords:** Coordinated medical care, Long-term care homes, Computerized documentation system, Hospital admissions, Complex intervention

## Abstract

**Background:**

Deficits in general and specialized on-site medical care are a common problem in nursing homes and can lead to unnecessary, costly and burdensome hospitalizations for residents. Reasons for this are often organizational obstacles (such as lack of infrastructure or communication channels) and unfavorable compensation structures, which impede the implementation of adequate medical care. The purpose of this study is to evaluate a complex intervention aiming to improve the coordination of medical care in long-term care nursing homes in Germany. The project aims to optimize the collaboration of nurses and physicians in order to reduce avoidable hospital admissions and ambulance transportations.

**Methods/design:**

In a prospective controlled trial, nursing home residents receiving a complex on-site intervention are compared to residents receiving care/treatment as usual. The study will include a total of around 4000 residents in approximately 80 nursing homes split equally between the intervention group and the control group. Recruitment will take place in all administrative districts of Baden-Wuerttemberg, Germany. The control group focuses on the administrative district of Tuebingen. The intervention includes on-site visits by physicians joined by nursing staff, the formation of teams of physicians, a computerized documentation system (CoCare Cockpit), joint trainings and audits, the introduction of structured treatment paths and after-hours availability of medical care. The project evaluation will be comprised of both a formative process evaluation and a summative evaluation.

**Discussion:**

This study will provide evidence regarding the efficacy of a complex intervention to positively influence the quality of medical care and supply efficiency as well as provide cost-saving effects. Its feasibility will be evaluated in a controlled inter-regional design.

**Trial registration:**

WHO UTN: U1111–1196-6611; DRKS-ID: DRKS00012703 (Date of Registration in DRKS: 2017/08/23).

**Electronic supplementary material:**

The online version of this article (10.1186/s12913-019-4156-4) contains supplementary material, which is available to authorized users.

## Background

With demographic aging in full force, an increasing number of elderly people are being cared for in nursing homes (NHs). Recent reports have indicated an alarming lack of primary on-site care provided in such facilities [1–4], the result of which can be unnecessary, costly and burdensome hospitalizations for residents [2, 5–10]. Previous studies have assessed that a fair amount of hospitalizations could be avoided by expanding on-site primary care [3–5, 11–13]. However, organizational obstacles such as lack of infrastructure or communication channels and unfavorable compensation structures often impede the implementation of adequate medical care [2, 3, 14]. This situation presents an increasing challenge to German physicians as they struggle to coordinate with one another [3, 13] and NH staff [3–5, 7, 13], hence establishing the need for a more systematic basis for cooperation. Interventions that might improve teamwork within groups of multidisciplinary NH care providers include advancing communication, regularly scheduled physicians’ NH visits (that are more appropriately compensated), after-hours availability and reducing administrative workload [10, 14–16]. Some of these have been proven to be effective in pilot projects [1, 2, 7].

Project CoCare (coordinated medical care) aims to improve the coordination of medical care in long-term care NHs by optimizing the collaboration of nursing staff and physicians in order to reduce the number of avoidable hospital admissions and ambulance transportations, which should improve quality and cost-effectiveness of medical care in long-term care NHs.

Additional or altered services in the project’s intervention include: on-site visits by physicians joined by nursing staff, the formation of teams of physicians, a computerized documentation system called CoCare Cockpit (CCC), joint trainings and audits, the introduction of structured treatment paths and after-hours availability of medical care.

The intervention is expected to positively influence quality of care and supply efficiency as well as provide cost-saving effects. The residents of long-term care NHs are surveyed about their health status, the perceived quality of medical care and the collaboration between physicians and nursing staff, perceived care continuity and quality as well as satisfaction with various care services. Nursing staff and physicians are asked to assess their collaboration as well as the continuity and quality of care provided.

The project evaluation is comprised of a formative process evaluation and a summative evaluation, with the latter being a control group design. The combination of process and summative evaluation complies with the recommendations for evaluating complex interventions [17]. In the intervention group, the concept outlined above is implemented and individual medical services administered are reimbursed according to a project-specific compensation plan. The intervention group includes all administrative districts of the federal state of Baden-Wuerttemberg, Germany except the administrative district of Tuebingen. The control group receives care/treatment as usual and includes the administrative district of Tuebingen, Baden Wuerttemberg. Each group will consist of approximately 40 NHs (for a total of 80) and include approximately 2000 long-term care home residents per group.

### Research aims

In this paper, we present the study protocol of project CoCare, developed as a nursing home-based intervention to improve the coordination of medical care in long-term care NHs. Additionally, the study aims a) to optimize the collaboration of nursing staff and physicians in order to b) reduce the number of avoidable hospital admissions and ambulance transportations.

## Methods/design

### Study design and setting

**Fig. 1:**
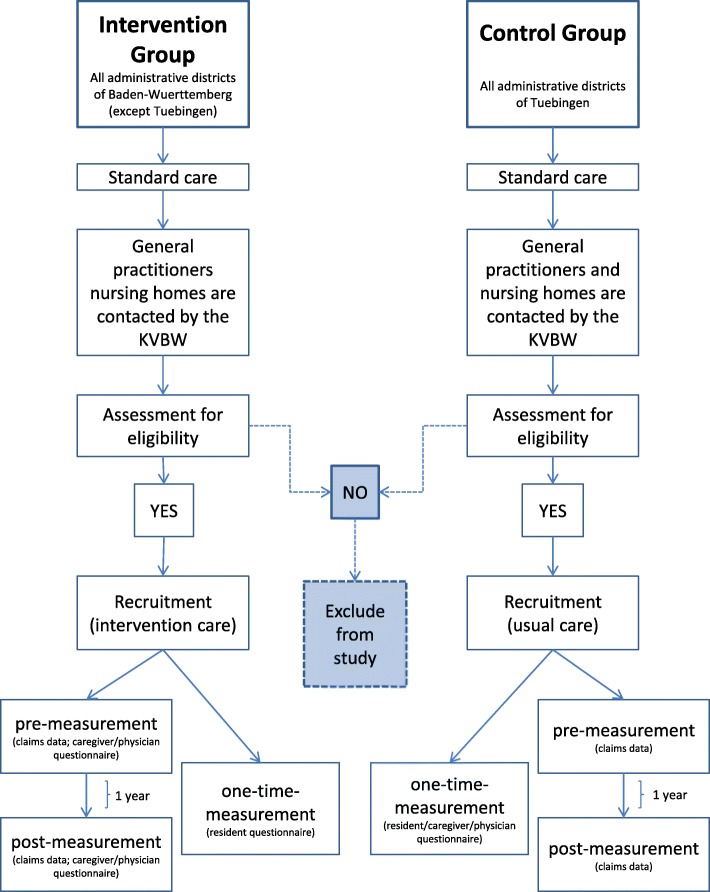
Study design (File attached); KVBW: Association of Statutory Health Insurance Physicians Baden Wuerttemberg

In a prospective controlled trial, the nursing home-based intervention will be tested by comparing an intervention group to a control group (Fig. 1). NHs for the intervention group will be recruited in all administrative districts of Baden-Wuerttemberg, except the administrative district of Tuebingen. NHs in the administrative district of Tuebingen, which was chosen because of its representativeness, will comprise the control group. Each group will include urban and rural districts to ensure that the results can be generalized to regions with different structural conditions (e.g. availability of medical care, long-term care NH structure).

### Intervention

The following processes and arrangements are introduced in the intervention groups:

A team of general practitioners (GP) looks after the residents of a NH and coordinates involvement of specialists. Weekly on-site visits take place at fixed times and are joined by nursing staff. Patients are assigned to their designated GP. However, GPs may treat any patient on behalf of another GP and can be reached by phone after office hours.

There are regular visits of specialists, at least quarterly, coordinated by GPs and accompanied by nursing staff. The residents visited are selected by GPs. In important cases, specialized physicians and GPs will try to coordinate their visits to the same day. Additionally, the project supports positioning suprapubic catheters in the NH, not only by offering training courses for physicians, but also by providing a transportable sonography device for each NH if necessary.

Standards and structured processes are facilitated between physicians and nursing staff. This includes structured workflows for unplanned cases, e.g. managing a crisis, as well as coordinating a consultation with physicians to prevent hospitalization. For this reason, treatment procedures (e.g., regarding pain) are structured and developed to involve all specialists and GPs.

To expand on usual medical care, the intervention includes coordinated medication management. Medication plans are written by GPs and monitored quarterly. For issues known to lead to frequent hospitalization of patients, structured preventive measures will be established and supported by checklists and action guidelines.

The project aims to improve communication and collaboration between physicians and nursing staff. This will be achieved by appointing study coordinators (CoCare study coordinators) at each participating NH as designated points of contact for physicians. CoCare coordinators are in charge of tasks such as documentation, preparation and follow up of on-site physician visits, etc.

In the intervention group individual medical services administered are reimbursed according to a project-specific compensation plan. Physicians are reimbursed for trainings and individual services, including coordinative activities, better reachability, or activities preventing hospitalization. Nursing homes receive a flat fee.

### Nursing homes

#### Eligibility criteria and recruitment

NHs that meet the following criteria are eligible to participate as a study site for the intervention group:

- Agree to install a secure internet connection that enables the use of the computerized documentation system CCC
- Collaboration with a team of GPs participating in the study
- Authorization according to Article 72 of Volume XI of the Social Insurance Code (Elftes Buch Sozialgesetzbuch - SGB XI)

The eligibility criteria for NHs in the control group are only the authorization according to Article 72 of Volume XI of the Social Insurance Code. GPs, who want to participate in the study, have to be willing to build a team with other GPs.

NHs and GPs were contacted and informed about the project by the Association of Statutory Health Insurance Physicians Baden Wuerttemberg (KVBW). NHs which met the criteria were invited to join the study. Based on their location, the NHs were assigned to either intervention or control group.

#### Training for nursing staff and physicians

One-day intensive training courses will optimize collaboration between physicians and nursing staff. To ensure structured and coordinated medical care, the courses are attended by both nursing staff and physicians and are based on treatment pathways developed by the Centre for Geriatric Medicine and Gerontology (ZGGF), Freiburg, Germany using recent literature [18, 19]. Since the project supports the correct changing of a catheter within an instructed time, the ZGGF also offered training courses for physicians in this regard.

Both nursing staff and physicians undergo additional training to work with the newly developed computerized documentation system named CoCare Cockpit (CCC). CCC is a web-based application that was developed by the nubedian GmbH for easy documentation and improved information and data management.

### Participants

#### Sample size

The study aims to include about 2000 residents in approximately 40 NHs in both the intervention and control group. In addition, 160 NH residents or relatives of NH residents and 80 nurses/physicians will participate in focus group interviews.

Using the Power and Sample Size Calculation software “Power and Precision” version 2.0 (Biostat), 253 participants are required per study group (80% power sample and *p* = .05; not considering potential dropouts) [20]. Because of the cluster design, it is necessary to calculate the design effect [21]. For a non-randomized controlled trial with approximately 50 observations in each cluster and intra-cluster correlation of *ρ* = 0.01, the design effect is calculated using the following formula:

DE = 1 + 0.01 × (50–1) = 1.49.

Therefore, a total of 377 cases (1.49 * 253) in both the intervention and control group are necessary. To allow for potential dropouts, *n* = 2000 participants were included in each group, since there is considerable uncertainty in the estimates. Thus, an unknown rate of missing values will most likely not affect quality of statistical analysis.

#### Recruitment of participants

NH residents, being potential participants, are contacted by their NH staff or their physician. Those who agree to join the study are assigned to either intervention or control group, based on the location of their NH. Exclusion criteria included dementia (for surveys and focus groups) and a residence time less than 3 months (Table 1).

**Table 1:** Participant eligibility criteria for residents

Inclusion	Exclusion
•Aged ≥18 years old	•Dementia (only for surveys and focus groups)
•Resident of a nursing home in an administrative districts in Baden Wuerttemberg	•Residence time in the long-term care home below the minimum time of 3 months
•Member of a statutory health insurance fund	

In the intervention group, coordinated medical care as outlined above is implemented. The intervention includes: establishing medical teams; a computerized documentation system named CoCare Cockpit (CCC); joint training courses and audits; joint on-site visits; annual meetings of all participating physicians and NH coordinators; quarterly meetings between GPs and NH coordinators; interdisciplinary, indication-specific case conferences as needed; standards and structured processes between the physician team and nursing staff; coordinated medication management; structured preventive measures; and extended availability of physicians. At baseline, study eligibility is screened for and informed consent of each participant is obtained in writing by the nursing staff before the recruited resident completes any questionnaire.

The control group participant receives care/treatment as usual based on the established care practices in their NH. At baseline, following the same inclusion/exclusion criteria as the intervention group (Table 1), informed consent is obtained in writing by the nursing staff before the recruited participant completes any questionnaire. Questionnaires will be provided by the nursing staff in each individual NH. Basic demographic data is assessed for comparison between participants of control and intervention group.

### Study measurements and outcomes

#### Study measurements

The evaluation is comprised of a formative process evaluation and a summative evaluation, with the latter being a control group design (Table 2).

**Table 2:** Summary of measurements and study outcomes

Measurements	Instruments	Group	Data Source	T0	in between	T1	Data analyses
Process evaluation							
Assessment of the intervention process from the view point of a resident	Focus group A	Intervention group	Resident		x		Qualitative analyses
Assessment of the intervention process from the view point of a caregiver/physician	Focus group B	Intervention group	Caregiver/physician		x		Qualitative analyses
Documentation of the implementation of pivotal intervention processes	Claims data	Intervention group	Health insurance company		x		Descriptive analyses
Organisational quality experienced the caregiver	Telephone interviews	Intervention group	Caregiver	x	x		Qualtitative and descriptive analyses
Summative evaluation							
Cost-based claims data from the German system of statutory health insurance (GKV)	Claims data	Intervention/control group	Health insurance company	x		x	Healthcare cost analyses, cost benefit analyses, cost-impact-analyses; propensity score adjustment
Audits and Peer-assessmen with caregivers							
a) Medical Quality	Audits and Peer-assessment of resident documens	Intervention group	Caregiver/physician		x		Qualtitative and descriptive analyses
b) Organizational Quality	Telephone interviews	Intervention group	Caregiver	x	x		Qualtitative and descriptive analyses
Quality experienced by the resident	Questionnaire	Intervention/control group	Resident		x		Comparison of intervention and control group using propensity score adjustment
Quality experienced by the caregiver/physician	Questionnaire	Intervention/control group	Caregiver/physician	x (intervention group)	x (control group)	x (intervention group)	Comparison of intervention and control group

T0 = baseline; T1 = 1 year after baseline

##### Process evaluation

Process evaluations aim to describe and assess all processes that are relevant to the success of the project. Knowledge gained will be reported back to all involved parties. Main questions of the process evaluation are:

- To what extent are the intervention elements (e.g., documentation of medication in CCC) put into practice?
- What intervention elements were not implemented and for what reasons?
- What contextual conditions were facilitating/impeding a positive outcome?
- What are the effective mechanisms of the intervention? Which intervention elements are most important?

This evaluation includes focus group interviews, descriptive evaluation of insurance claims data and telephone interviews.

Focus group interviews are used to assess the processes implemented in the intervention. Focus group A, conducted with NH residents and relatives, discusses aspects of implemented care (e.g., joint visits by physician and nursing staff). Focus group B, including nursing staff and physicians, focuses on administrative aspects such as interdisciplinary, indication-specific case conferences (see also Additional files 1 and 2).

Cost-based claims data from the German system of statutory health insurance is used to assess all elements of the intervention involving residents. This includes aspects such as the completion of on-site visits and medication management.

Non-patient related, organizational aspects of the intervention are assessed by telephone interviews (see also Additional file 3). Interviews with the CoCare study coordinators at each NH are conducted quarterly. Questions asked during the interviews include: “Did the nursing home appoint a CoCare study coordinator as contact person for GPs?”; “Did the project coordinator of the nursing home organize the on-site visit by the physician?”

The interviews will be used to assess adverse events and will enable the project coordinator to intervene if necessary. Interviews are conducted in both process evaluation and summative evaluation.

##### Summative evaluation

The summative evaluation focuses on the effects of the intervention in terms of its quality and cost indicators compared to the control group.

On an individual level, the following cost indicators will be assessed via claims data: 1. Total cost of hospitalization; 2. Total cost of patient transport; 3. Total cost of outpatient treatments by GPs; 4. Total cost of outpatient treatments by medical practitioners; 5. Total cost of medication and medical supplies; 6. Assessment of additional cost for measures implemented in the intervention group. Cost-based claims data (indicators 1–5) is provided by the German system of statutory health insurance. Information about additional costs is gathered through intervention-related billing data (indicator 6). All claims data in the intervention and control group was pseudonymized and anonymized, respectively.

Regarding peer assessment, the quality of medical, patient related procedures will be evaluated by the ZGGF via audits. Patient related procedures include correct changing of a catheter within an instructed time. Non-patient related, organizational aspects of the intervention are assessed via telephone interviews by the Section of Health Care Research and Rehabilitation Research, Faculty of Medicine and Medical Center - University of Freiburg (see also Additional file 3). Questions include: “Are on-site visits by specialized physicians accompanied by assigned nursing staff?”

Furthermore, residents will be questioned about additional indicators of quality, including perceived quality of care (with a focus on medical care provided by GPs and cooperation of GPs and nursing staff), overall satisfaction with care in the NH, perceived state of health and overall quality of life. Since no available questionnaire is suitable for this particular intervention, a questionnaire assessing perceived quality of care will be developed. A number of instruments regarding overall satisfaction with care processes are available internationally [22]. However, only few validated and reliable measures are available in German. Hence, a modified version of the commonly used questionnaire “ZUF-A-7” [23] will be used in this study. Perceived state of health and overall quality of life is assessed using the (nationally/internationally) established World Health Organization Quality of Life Questionnaire - WHOQOL-OLD [24] (see also Additional file 4).

Nursing staff are instructed to motivate residents to fill out all questionnaires and assist in case of lack of clarity of questions. However, no additional assistance (such as filling out forms on behalf of a resident or prompting answers) by nursing staff is allowed. In case residents do require additional assistance, residents’ relatives may be asked to assist filling out questionnaires. Any such assistance is to be documented on the questionnaire.

In addition to patient related processes, the intervention also includes organizational aspects that can only be monitored by nursing staff (e.g., the occurrence of interdisciplinary, indication-specific case conferences). Therefore, nursing staff and physicians will also complete a questionnaire (see also Additional files 5, 6, 7 and 8). Questions are based on work by Körner and Wirtz [25] assessing perceived teamwork of nursing staff and physicians, as well as a questionnaire regarding the working conditions of physicians by Fischbeck and Laubach [26] will be included and address physicians and nursing staff. Data will be collected at two time points: baseline (t0) and 12 months later (t1; Table 2). The control group will be questioned once. All questionnaire data will be assessed anonymously.

#### Study outcomes

Primary outcomes of the study are as follows: a) health economic analyses, including total cost of health care (assessment of cost related claims data of the statutory health insurance; pre-post measurement); b) quality of care analyses, including quality of care experienced by residents, physicians and nursing staff (questionnaires for residents, physicians and nursing staff; audits). Data will be included for the period of January 1, 2017 until September 30, 2020.

#### Hypotheses

The hypothesis for health economic analyses is that the total cost of health care will be lower in the intervention group compared to the control group. Regarding quality of care, the hypothesis for patient reported outcomes is that perceived quality of care as well as overall satisfaction with received care in the NH is significantly better in the intervention croup compared to the control group, even after adjusting for relevant confounding variables. The hypotheses for nursing staff/physicians reported outcomes are that a) the perceived quality of medical/nursing care is significantly better in the intervention group compared to the control group; and b) the perceived quality of medical/nursing care is evaluated significantly better after conducting the intervention compared to baseline.

#### Data analysis

Data will be collected from claims data, audits, telephone interviews, focus groups, and questionnaires (residents, nursing staff and physicians).

Claims data will be used to conduct economic analyses. It will be evaluated to what extent the claiming of benefits (indicators 1–5) differs between both groups regarding time and certain characteristics of the residents (age, gender, care level/care degree, dementia). Subsequently, cost indicators will be aggregated on a residential level. The resulting sum will equate the overall cost of the medical benefits claimed, including project costs. With a cost-benefit analysis, it will be examined to what extent the total cost differs between intervention and control group. Remaining differences between the two groups will be addressed using propensity score adjustment [27]. Results may also be used for budget-impact-analyses from a health insurance company’s perspective. Lastly, quality of life will be linked to the total cost of medical care with a cost-effectiveness analysis. Thereby, improvements in quality indicators such as ‘perceived quality of care’, ‘overall satisfaction with care’, ‘perceived state of health’ and ‘quality of life’ will be analyzed in relation to the costs of medical resource utilization by calculating incremental cost effectiveness ratios.

Because health care costs are not normally distributed, a generalized linear model (GLM) with a gamma distribution and log link transformation will be used for data analysis [28]. To account for the multi-level-structure of the data, a random intercept will be added on the regional or NH level. Finally, regression estimates will be converted back to costs for interpretation. Incremental cost effectiveness ratios and corresponding confidence intervals will be estimated using seemingly unrelated regressions [29].

For the peer assessment, the basis of valuation for the audits will be based on data in the CCC. For non-patient related organizational aspects of the intervention, data will be collected in telephone interviews. Both analyses will be integrated in the report.

Focus group interviews will be recorded. The content-analytical evaluation of the audio recordings will be based on an approach by Mayring [30] using the ATLAS.ti software [31]. The sample size was chosen based on experiences with previous multicenter studies.

Data collection by questionnaires will apply to residents as well as nursing staff and physicians. Due to numerous factors, the intervention will probably improve residents’ quality of life to a lesser extent than quality of care. For this reason, no clear statistically significant superiority is postulated. However, quality of life will be included in the cost-effectiveness analysis to control for unexpected effects of the intervention. In accordance with previous intervention studies, a non-responder rate of 60% is assumed [32, 33]. The statistical analysis strategy is the same used with cost data (GLM; taking into consideration the multi-level-structure as well as propensity score adjustment). It is essential to account for the multi-level-structure, since differences between facilities are to be expected. For nursing staff and physician questionnaires, a non-responder rate of 30% is assumed. Subgroup analyses will control for potential differences between professional/occupational groups.

Data will be analyzed using IBM SPSS Statistics for windows [34], MPlus [35] and Stata [36]. Throughout the study, alpha levels will be fixed at α = 0.05 for all statistical tests.

## Discussion

Exploring and developing new interventions to improve living conditions of residents of long-term care NHs is crucial. Aside from detrimental effects on individual quality of life, the cost of long-term care NHs has become an increasing financial burden on society. Since a considerable number of hospitalizations could be avoided, this project will determine if improved coordination of medical care and an optimized collaboration of nursing staff and physicians can reduce the number of hospital admissions and ambulance transportations. To our knowledge, this study is the first to develop a complex intervention to positively and efficiently influence quality and cost of care in NHs as well as evaluate the intervention’s feasibility in a controlled design.

## Additional files

Additional file 1: Interview guide focus group– residents. (PDF 78 kb)

Additional file 2: Interview guide focus group– medical staff. (PDF 78 kb)

Additional file 3: Interview guide process evaluations. (PDF 99 kb)

Additional file 4: NH resident questionnaire. (PDF 338 kb)

Additional file 5: Doctor questionnaire baseline. (PDF 294 kb)

Additional file 6: Nursing staff questionnaire baseline. (PDF 264 kb)

Additional file 7: Doctor questionnaire follow-up. (PDF 311 kb)

Additional file 8: Nursing staff questionnaire follow-up. (PDF 300 kb)

## Abbreviations

CCC: CoCare Cockpit
CoCare: Coordinated medical care
GLM: Generalized linear models
GP: General practitioner
KVBW: Association of Statutory Health Insurance Physicians Baden Wuerttemberg
NH: Nursing home
ZGGF: Centre for Geriatric Medicine and Gerontology
