# Supplementary material for: A complex health services intervention to improve medical care in long-term care homes: study protocol of the controlled coordinated medical care (CoCare) study
Source: BMC Health Serv Res. 2019 May 24;19:332. doi: 10.1186/s12913-019-4156-4 (PMC6534891; doi:10.1186/s12913-019-4156-4)
Supplement: Supplementary file 1 — Interview guide focus group– residents. (PDF 78 kb) [file 12913_2019_4156_MOESM1_ESM.pdf]

# CoCare interview guide for resident focus groups

## Guiding questions

**I'd like to ask you a few questions that refer to the time since** *[date of interven-tion begin],* **which is the date that this project started. I'd like to know if anything has changed for any of you personally.**

| | |
|---|---|
| | First, here are some questions concerning your **general practitioner/ primary care doctor.** |
| 1. | Please think back to *[date of intervention begin].* How often has your general practitioner come to see you? *(Explain as needed: weekly, biweekly, monthly, infrequently...?)* |
| 2. | When thinking of the time before *[date of intervention begin],* has the frequency of doctor's visits changed? |
| 3. | When you need medical attention, does your primary care doctor come to see you? Has that been the case since before *[date of intervention begin]*? |
| 4. | Did your primary care doctor ever visit you after office hours when necessary (e.g. after you suffered a fall or similar)? Has that been the case since before *[date of intervention begin]*? |
| 5. | When your doctor is doing rounds, are nursing staff attending the visits? Has that been the case since before *[date of intervention begin]*? |
| 6. | Did you have a single, assigned primary care doctor? Or did different doctors come to see you? |
| | *Halftime: Please check the time to see if you're on schedule* |
| | The next few questions concern the care you receive from **special-ists/specialized doctors, like urologists, dermatologists or neurologists**. |
| 7. | When thinking back to *[date of intervention begin],* how often has a specialist come to consult with you since? *(Explain as needed: at least quarterly, more infrequently...?)* |
| 8. | When thinking of the time before *[date of intervention begin],* has the frequency of specialists' visits changed? |
| 9. | Are there any doctors, specialized or not, that have started coming to visit you (as opposed to you visiting them at their office) since *[date of intervention begin]*? |

| | |
|---|---|
| | The following question will concern the way in which doctors and nursing staff **talk to each other.** |
| 10. | Is it your impression that communication between doctors and nursing staff has improved since *[date of intervention begin]*? |
| | This question refers to the **medication** you receive. |
| 11. | Since *[date of intervention begin], do* you feel like your medication has been adjusted properly to fit your needs and account for other medication you were prescribed? Have issues concerning your medication subsided, like adverse side effects? Or did you notice no differences? |
| | Lastly, I'd like to ask a **more general** question. |
| 12. | Do you feel like the medical care you receive at your nursing home has improved since *[date of intervention begin]*? If so: What specifically made you feel like your care has improved? |
