# Supplementary material for: A complex health services intervention to improve medical care in long-term care homes: study protocol of the controlled coordinated medical care (CoCare) study
Source: BMC Health Serv Res. 2019 May 24;19:332. doi: 10.1186/s12913-019-4156-4 (PMC6534891; doi:10.1186/s12913-019-4156-4)
Supplement: Supplementary file 2 — Interview guide focus group– medical staff. (PDF 78 kb) [file 12913_2019_4156_MOESM2_ESM.pdf]

CoCare interview guide for medical staff focus groups

Attendees were asked to choose which of the following topics would be most relevant to the group before starting the interview process.

Shared patient medical record / CoCare-Cockpit

1) Did you use CoCare-Cockpit? If so: To what extent was it used?
2) What **benefits** did you experience in using CoCare-Cockpit?
3) What factors **facilitated** the use of CoCare-Cockpit?
4) What **barriers** did you need to overcome in using CoCare-Cockpit?
5) Is there anything you'd like to **add** regarding CoCare-Cockpit?

Rounds

6) What **changes** did you observe regarding rounds after project CoCare was implemented?
   a) Were rounds **regularly scheduled** (preferably weekly)?
   b) Did **organization** of rounds improve?
   c) Were **responsibilities assigned clearly?**
7) What **benefits** did you experience with CoCare-rounds?
8) What **helped you** implement CoCare-rounds?
9) Which **barriers** did you have to overcome to implement CoCare-rounds?
10) Is there anything you'd like to **add** about CoCare-rounds?

General practitioner teams

11) How useful did you find the medical care provided by **GP-teams?**
12) Which factors **facilitated** the consolidation of GP-teams?
13) Which factors **impeded** the consolidation of GP-teams?
14) What are the **advantages/disadvantages** of GP-teams?
15) Is there anything you'd like to **add** about GP-teams?

Medication checks

16) Did you perform medication checks on a regular basis?

17) *If so:* What factors **facilitated** the implementation of medication checks? How **useful** did you find the medication checks?

18) *If not:* Why weren't regularly scheduled medication checks performed?

Communication and collaboration doctors-nursing staff

19) How did the **collaboration between doctors and nursing staff** change since project CoCare?

20) How did the **collaboration between doctors** change since project CoCare?

21) How did the **collaboration between general practitioners and specialists** change since project CoCare?

22) What factors of project CoCare **facilitated** collaboration?

23) What factors of project CoCare **impeded** collaboration?

24) Did you hold quarterly and yearly meetings?

25) *If so:* What factors **facilitated** the implementation of quarterly or yearly meetings? How would you rate the **usefulness** of those meetings?

26) *If not:* Why were no quarterly or yearly meetings held?

Extended availability

27) Was the availability of GPs by phone extended until at least 9 p.m. on weekdays?

28) *If so:* What factors **facilitated** the implementation of extended availability? How **useful** did you find the extended availability?

29) *If not:* Why was extended availability not implemented?

Case conferences

30) Were case conferences held, if applicable?

31) *If so:* What factors **facilitated** the implementation of case conferences? How **useful** did you find them?

32) *If not:* Why were no case conferences held?

Standard courses of treatment / Training courses

33) How would you rate the off-site training for standard courses of treatment within the project?

34) Did you implement the CoCare-recommended standard courses of treatment at your facility?

35) *If so:* What factors **facilitated** the implementation of CoCare's standard courses of treatment? What **benefits** did you experience?

36) *If not:* Why were CoCare's standard courses of treatment not implemented?

Please ask in each focus group interview (allow for approximately 20 min before ending the interview):

Overall assessment

- Would you like for the CoCare-concept to be part of standard care in nursing homes?
- Which contextual factors would **facilitate,** which **impede** the implementation of the concept?
- What **benefit**s do you see in implementing the concept?
- Did **medical care provided to residents** of the facility improve, e.g. by avoiding hospitalizations?
- What specific measures of CoCare were **especially important** and why?
- What **disadvantages** did you experience?
- What **modifications** would you make to the concept?
