# Supplementary material for: A complex health services intervention to improve medical care in long-term care homes: study protocol of the controlled coordinated medical care (CoCare) study
Source: BMC Health Serv Res. 2019 May 24;19:332. doi: 10.1186/s12913-019-4156-4 (PMC6534891; doi:10.1186/s12913-019-4156-4)
Supplement: Supplementary file 3 — Interview guide process evaluations. (PDF 99 kb) [file 12913_2019_4156_MOESM3_ESM.pdf]

CoCare
coordinated medical care

Interview guide for process evaluations

Quarter: ___________________________

Instructions
Your answers to the following questions will be utilized to assess project CoCare's processes (i.e. „process evaluation“). We are interested to know how well the implementation of the project is going in your facility, whether there were any issues in implementing the project and if so, to specify those issues. The results obtained in these interviews will be used for internal discussions within the CoCare project team, aiming to improve CoCare processes.<br>All questions refer to the **last quarter,** i.e. (for example) **January to March 2018.**

1. Nursing home coordinators

Did you instate **one nursing home coordinator** as well as **1-2 proxies** to be fixed persons of contact for all enrolled doctors?

☐ Yes ☐ No

✍ ______________________________

____

Did nursing home coordinators collaborate with enrolled doctors to agree on a **timetable for regularly scheduled doctor's visits**?

☐ Yes ☐ No

If not:

Who coordinated timetables for doctor's visits?

✍ ______________________________

✍ ______________________________

____

Did nursing home coordinators **organize nursing staff attendance during rounds**?

☐ Yes ☐ No

If not:

Who organized nursing staff attendance during rounds?

✍ ______________________________

✍ ______________________________

____

Did nursing home coordinators **prepare doctor's rounds** (e.g., notify doctors of high risk patients, prepare CoCare-Cockpit documentation)?

☐ Yes ☐ No

If not:

Who did prepare rounds?

✍ ______________________________

✍ ______________________________

____

Did nursing home coordinators take on **documentation in CoCare-Cockpit, the joint patient medical records platform**?

☐ Yes

☐ No

If not:

Who did take on documentation in CoCare-Cockpit?

✍ ______________________________

✍ ____

____

Did nursing home coordinators ensure the **implementation of doctor's orders**?

☐ Yes

☐ No

If not:

Who ensured the implementation of doctor's orders?

✍ ______________________________

✍ ____

____

Overall, how would you rate the organization of different processes within the project?

☐	☐	☐	☐	☐	☐
1<br>very good	2<br>good	3<br>adequate	4<br>sufficient	5<br>poor	6<br>very poor

✍ ____

____

2. Teams of general practitioners

Did a team of 2-5 GPs (depending on the facility's size: more than one team) **jointly provide medical care** for the nursing home residents?

☐ Yes ☐ No

✍ ______________________________

____

Did GPs choose which **patients to refer to specialists?**

☐ Yes ☐ No

If not:

Who chose patients to refer to specialists?

✍ ______________________________

✍ ______________________________

____

Overall, how would you asses the medical care provided to your facility by GPs and specialists? What went well, what was difficult?

✍ ______________________________

____

____

____

____

____

____

____

3. Communication

In the last quarter, did you hold a **joint meeting between GPs and nursing home coordinators**, or alternatively, a **joint meeting of all enrolled doctors and nursing home coordinators**?

☐ Yes ☐ No

✍ ______________________________

____

If applicable, were any **interdisciplinary, indication-specific case conferences** held, perhaps including relatives or powers of attorney (POA)?		
☐ Yes<br><br>If so:<br>How many case conferences were held?<br>✍ ____________	☐ No	☐ Not applicable
✍ ____________		

**Overall,** how would you assess communication between doctors and nursing staff? What went well, what was difficult?
✍ ____________

4. Medical care

Did you confront issues that often lead to hospitalizations with **preventative measures** derived from **checklists and guidelines**?

☐ Yes

If so, were those checklists and guidelines...

☐ **...facility-specific checklists** or **expert standards of care?**

☐ **...CoCare-recommended standard courses of treatment?**

☐ No

✍ ____

____

Was a GP within the GP-team available by phone **after office hours** (Monday through Friday **until at least 9p.m.**)?

☐ Yes

☐ No

✍ ____

____

Please imagine the following situation:

You have a reasonable suspicion one of your residents might need emergency medical care and no GP is available, which might lead to the **hospitalization of the resident**.

Please assess: Has this situation changed since **CoCare interventions have been implemented?**

This situation has...

☐ …improved

☐ …deteriorated

If the situation has improved or deteriorated, where do you see the cause for that change?

✍ ____

____

☐ ….not changed

✍ ____

____

5. Overall project
Is there anything **you'd like to add** concerning processes or organization in project CoCare?
✍ ________________________________

Thank you for taking the time to answer these questions!
