# Supplementary material for: A complex health services intervention to improve medical care in long-term care homes: study protocol of the controlled coordinated medical care (CoCare) study
Source: BMC Health Serv Res. 2019 May 24;19:332. doi: 10.1186/s12913-019-4156-4 (PMC6534891; doi:10.1186/s12913-019-4156-4)
Supplement: Supplementary file 4 — NH resident questionnaire. (PDF 338 kb) [file 12913_2019_4156_MOESM4_ESM.pdf]

Project
Extended coordinated medical care in nursing homes
(CoCare)

Questionnaire for nursing home residents

Section of Health Care Research and Rehabilitation Research

Please turn over

Instructions for completing the questionnaire

In this questionnaire, we'd like to know more about your nursing home and the medical care you receive there. Other topics will cover how you perceive your health and quality of life, as well as some general personal questions. Completing the questionnaire will take about 30 minutes.

Please answer these questions one after another in the sequence they appear on the questionnaire. **Please don't skip any questions.**

If you have trouble filling out the questionnaire by yourself, feel free to ask a relative or another person to help you. However, this person shouldn't influence your answers in any direction. We ask that nursing staff or doctors do not help you complete this questionnaire, as we would like to know what **you** think.

- ✓ You will answer most of the questions by checking a box. Please **check only one box per question.**
- ✓ **Please choose one of the options provided**! For some questions, you may need to fill in dates, numbers or words.
- ✓ If you feel like a question doesn't apply to you, **please check a box regardless**. Most of the time, one of the options provided will be an appropriate answer, e.g. "don't know" or "doesn't apply".
- ✓ A few questions will be very similar to one another, since this questionnaire consists of several shorter questionnaires. **Please answer all the questions regardless of repetition.**
- ✓ In case you need to correct an answer (i.e. you accidently checked the wrong box), please do so by filling in the falsely checked box completely. You may now check the correct box:

**<u>Example</u> question:**	strongly agree	agree	agree some-what	disagree some-what	dis-agree	strongly dis-agree t
I actively participated in the discussion.	☐	■	☒	☐	☐	☐

Thank you for taking the time to participate in this survey!

Evaluate your nursing home

The following questions will ask you to assess the cooperation between doctors and nursing staff in your facility.

Please indicate to which extent the following statements apply to you.

	don't know	strongly disagree	disagree	agree	strongly agree	
	☐	☐	☐	☐	☐	1
						2
	☐	☐	☐	☐	☐	3

Questions 1 through 3 are adapted from a questionnaire assessing work satisfaction for doctors (Fragebogen zur Arbeitssituation von Ärzten; FAÄ):

Fischbeck, S., & Laubach, W. (2005). Arbeitssituation und Mitarbeiterzufriedenheit in einem Universitätsklinikum: Entwicklung von Messinstrumenten für ärztliches und pflegerisches Personal. *PPmP-Psychotherapie· Psychosomatik· Medizinische Psychologie*, *55*(06), 305-314.

	don't know	strongly disagree	disagree	agree	strongly agree	
	☐	☐	☐	☐	☐	4
	☐	☐	☐	☐	☐	5
						6
						7
	☐	☐	☐	☐	☐	8
	☐	☐	☐	☐	☐	9
	1	2	3	4	5	

Questions 4 through 9 are adapted from the Internal Participation Scale (IPS):

Körner, M., & Wirtz, M. A. (2013). Development and psychometric properties of a scale for measuring internal participation from a patient and health care professional perspective. *BMC health services research*, *13*(1), 374.

With these next few questions, we'd like to see how satisfied you are with your facility and the care you receive there.

Please indicate to which extent the following statements apply to you.

	no	mostly no	mostly yes	yes	
	☐	☐	☐	☐	10
	☐	☐	☐	☐	11
	[illegible]	[illegible]	[illegible]	[illegible]	12
	[illegible]	[illegible]	[illegible]	[illegible]	13
	☐	☐	☐	☐	14
	1	2	3	4	

	very dissatisfied	mostly dissatisfied	mostly satisfied	very satisfied	
	☐	☐	☐	☐	15
	☐	☐	☐	☐	16
	1	2	3	4	

Questions 10 through 16 are adapted from a questionnaire assessing satisfaction of relatives with the medical care received in nursing homes (Zufriedenheit von Angehörigen mit der Versorgung in stationären Altenpflegeeinrichtungen; ZUF-A-7):

Kriz, D., Schmidt, J., & Nübling, R. (2006). Zufriedenheit von Angehörigen mit der Versorgung in stationären Altenpflegeeinrichtungen. Entwicklung des Screening-Fragebogens ZUF-A-7. *Pflege*, *19*(2), 88-96.

Please turn over

Assess your medical consultations

How would you describe your behavior while consulting with your doctors?

Passive might mean you wait for doctors to let you know what they want to speak about and not express concerns.

Active might mean you ask questions and express opinions and concerns.

- ☐ 1 Very passive 17
- ☐ 2 Mostly passive
- ☐ 3 Mostly active
- ☐ 4 Very active

The following questions concern the medical care you receive in your facility.

Are you able to see a general practitioner whenever necessary?

Yes	No	18
☐ 1	☐ 2	

Are you able to see a specialist whenever necessary? (Specialists are specialized doctors like neurologists, urologists, pain therapists or dermatologists.)

Yes	No	I don’t need to see a specialist.	19
☐ 1	☐ 2	☐ 3	

very dissatisfied	mostly dissatisfied	mostly satisfied	very satisfied	20
☐	☐	☐	☐	

Question 20 is adapted from ZUF-A-7, see citation above.

Please choose only *one* option per question.

	Not at all	A little	A moderate amount	Very much	An extreme amount	
	☐	☐	☐	☐	☐	21
	☐	☐	☐	☐	☐	22
	☐	☐	☐	☐	☐	23
	☐	☐	☐	☐	☐	24
						25
	☐	☐	☐	☐	☐	26
	☐	☐	☐	☐	☐	27
	☐	☐	☐	☐	☐	28
	☐	☐	☐	☐	☐	29
	1	2	3	4	5	

Questions 21 through 29 are the WHO Quality of Life questionnaire for the elderly (WHOQOL-OLD):

World Health Organization. (2006). WHOQOL-OLD Manual. *Copenhagen: World Health Organization*.

Please choose only *one* option per question.

	Not at all	A little	Moderately	Mostly	Completely	
	☐	☐	☐	☐	☐	30
	☐	☐	☐	☐	☐	31
	☐	☐	☐	☐	☐	32
	☐	☐	☐	☐	☐	33
	☐	☐	☐	☐	☐	34
	1	2	3	4	5	

Questions 30 through 34 are the WHOQOL-OLD, see citation above

Please turn over

Please choose only *one* option per question.						
	Very dissatisfied	Dissatisfied	Neither satisfied nor dissatisfied	Satisfied	Very satisfied	
	☐	☐	☐	☐	☐	35
	☐	☐	☐	☐	☐	36
	☐	☐	☐	☐	☐	37
	☐	☐	☐	☐	☐	38
	☐	☐	☐	☐	☐	39
	☐	☐	☐	☐	☐	40
	1	2	3	4	5	

Questions 35 through 40 are the WHOQOL-OLD, see citation above

Please choose only *one* option per question.						
	Not at all	A little	Moderately	Mostly	Completely	
	☐	☐	☐	☐	☐	41
	☐	☐	☐	☐	☐	42
	☐	☐	☐	☐	☐	43
	☐	☐	☐	☐	☐	44
	1	2	3	4	5	

Questions 41 through 44 are the WHOQOL-OLD, see citation above

Health

The following are questions about your health.

Choose one option per question.

In general, would you say your health is:

- [ ] 1 Excellent 45
- [ ] 2 Very good
- [ ] 3 Good
- [ ] 4 Fair
- [ ] 5 Poor

Compared to one year ago, how would you rate your overall health **now?**

- [ ] 1 Much better now than one year ago 46
- [ ] 2 Somewhat better now than one year ago
- [ ] 3 About the same
- [ ] 4 Somewhat worse now than one year ago
- [ ] 5 Much worse now than one year ago

Please turn over

Personal information

When reporting results of a project, we need to describe the participants of the study. In order to do that, we'd like to ask you the following personal questions.

When did you complete this questionnaire? 47

Date ☐☐ ☐☐ ☐☐☐☐

Your gender 48

☐ Male (1)

☐ Female (2)

Your nationality 49

☐ German (1)

☐ Other (2)

How old are you? 50

- ☐ 1 Under 59 years old
- ☐ 2 60 to 64 years old
- ☐ 3 65 to 69 years old
- ☐ 4 70 to 74 years old
- ☐ 5 75 to 79 years old
- ☐ 6 80 to 84 years old
- ☐ 7 85 to 89 years old
- ☐ 8 90 years or older

Do you have relatives that visit you? 51

- ☐ 1 Yes
- ☐ 2 No
- ☐ 3 I have no relatives

Please turn over

What is the highest degree or level of schooling you have completed?

- ☐ 1 No schooling completed 52
- ☐ 2 Grade school or Certificate of Secondary Education (9yrs of education)
- ☐ 3 General Certificate of Secondary Education (GCSE, 10yrs of education)
- ☐ 4 High school (former GDR equivalent of GCSE, 10yrs of education)
- ☐ 5 „Fachhochschulreife“, „Fachoberschule“ (Advanced technical college admissions certificate, 12 yrs of education)
- ☐ 6 General qualification for university entrance, „Abitur“ (12-13 yrs of education)
- ☐ 7 Other

How long have you been living in your facility?

- ☐ 1 Less than six months 53
- ☐ 2 More than six months, less than a year
- ☐ 3 One to two years
- ☐ 4 More than two years

Did anyone help you complete this questionnaire?

- ☐ 1 No one, I answered all questions and indicated all answers by myself. 54
- ☐ 2 Relative
- ☐ 3 Friend or acquaintance
- ☐ 4 Somebody else

Please specify: ____ 55

End of questionnaire.

Thanks so much for completing the questionnaire and participating in our survey!
