# Supplementary material for: A complex health services intervention to improve medical care in long-term care homes: study protocol of the controlled coordinated medical care (CoCare) study
Source: BMC Health Serv Res. 2019 May 24;19:332. doi: 10.1186/s12913-019-4156-4 (PMC6534891; doi:10.1186/s12913-019-4156-4)
Supplement: Supplementary file 6 — Nursing staff questionnaire baseline. (PDF 264 kb) [file 12913_2019_4156_MOESM6_ESM.pdf]

Project
Extended coordinated medical care in nursing homes
(CoCare)

Questionnaire for nursing staff

1st survey (baseline)

Intervention group

Section of Health Care Research and Rehabilitation Research

Instructions for completing the questionnaire

With the following questions, we'd like to know more about your work in the enrolled nursing home and how you experience the cooperation with nursing staff.

Please answer these questions one after another in the sequence they appear on the questionnaire. **Please don't skip any questions.**

- ✓ You will answer most of the questions by checking a box. Please **check only one box per question.**
- ✓ **Please choose one of the options provided**! For some questions, you may need to fill in dates, numbers or text.
- ✓ If you feel like a question doesn't apply to you, **please check a box regardless**. Most of the time, one of the options provided will be an appropriate answer, e.g. "don't know" or "doesn't apply".
- ✓ A few questions will be very similar to one another, since this questionnaire consists of several shorter questionnaires. **Please answer all the questions regardless of repetition.**
- ✓ In case you need to correct an answer (i.e. you accidently checked the wrong box), please do so by filling in the falsely checked box completely. You may now check the correct box:

**Example question:**	**strongly agree**	**agree**	**agree some-what**	**disagree some-what**	**dis-agree**	**strongly dis-agree**	**t**
I actively participated in the discussion.	☐	■	☒	☐	☐	☐	

Thank you for taking the time to participate in this survey!

Assessment of cooperation

The following questions concern the collaboration of all enrolled nursing staff and general practitioners/specialists involved in caring for nursing home residents. Please indicate to which extent the following statements apply to you.

Please refer your answers to the time **before CoCare was implemented** at your facility.

Please choose only *one* option per item.

	strongly disagree	disagree	agree	strongly agree	don't know	
	☐	☐	☐	☐	☐	1
	☐	☐	☐	☐	☐	2
	☐	☐	☐	☐	☐	3
	☐	☐	☐	☐	☐	4
	☐	☐	☐	☐	☐	5
	☐	☐	☐	☐	☐	6
	☐	☐	☐	☐	☐	7
	1	2	3	4	5	

Questions 1 through 7 are adapted from the Internal Participation Scale (IPS):

Körner, M., & Wirtz, M. A. (2013). Development and psychometric properties of a scale for measuring internal participation from a patient and health care professional perspective. *BMC health services research*, *13*(1), 374.

The following questions concern the collaboration with nursing staff at the facility you provide medical care for. Please choose one of the response options and indicate to which extent the chosen option applies to you.

Please refer your answers to the time **before CoCare was implemented** at your facility.

**very good**					**very poor**	8
☐	☐	☐	☐	☐	☐	
**1**	**2**	**3**	**4**	**5**	**6**	
1	2	3	4	5	6	

**very good**					**very poor**	**don't know**	9
☐	☐	☐	☐	☐	☐	☐	
**1**	**2**	**3**	**4**	**5**	**6**	**7**	
1	[illegible]	[illegible]	[illegible]	[illegible]	[illegible]	7	

Questions 8 through 14 are adapted from a questionnaire assessing work satisfaction for nursing staff (Fragebogen zur Arbeitssituation von Pflegekräften; FAP):

Fischbeck, S., & Laubach, W. (2005). Arbeitssituation und Mitarbeiterzufriedenheit in einem Universitätsklinikum: Entwicklung von Messinstrumenten für ärztliches und pflegerisches Personal. *PPmP-Psychotherapie· Psychosomatik· Medizinische Psychologie*, *55*(06), 305-314.

**very m[illegible]**					**very little**	10
☐	[illegible]	[illegible]	[illegible]	[illegible]	☐	
**1**	[illegible]	[illegible]	[illegible]	[illegible]	**6**	
1	2	3	4	5	6	

**very much**					**very little**	11
☐	☐	☐	☐	☐	☐	
**1**	**2**	**3**	**4**	**5**	**6**	
1	2	3	4	5	6	

very much					very little	12
☐	☐	☐	☐	☐	☐	
**1**	**2**	**3**	**4**	**5**	**6**	
1	2	3	4	5	6	

absolutely sufficient					absolutely insufficient	13
☐	☐	☐	☐	☐	☐	
**1**	**2**	**3**	**4**	**5**	**6**	
1	2	3	4	5	6	

always available					never available	14
☐	☐	☐	☐	☐	☐	
**1**	**2**	**3**	**4**	**5**	**6**	
1	2	3	4	5	6	

Evaluation of Care

The following statements concern the medical care of nursing home residents. Please indicate to which extent the statements apply to the facility you provide medical care for.

Please refer your answers to the time **before CoCare was implemented** at your facility.

Please choose only *one* option per item.

	excellent	very good	good	satis-factory	poor	very poor	
**Primary medical care** at our facility is…	☐	☐	☐	☐	☐	☐	15
Cooperation between doctors and nursing staff concerning patient care is…	☐	☐	☐	☐	☐	☐	16
**Specialized medical care** at our facility is…	☐	☐	☐	☐	☐	☐	17
Rounds at this facility are …	☐	☐	☐	☐	☐	☐	18
Documentation of rounds is…	☐	☐	☐	☐	☐	☐	19
The implementation of doctor's orders is…	☐	☐	☐	☐	☐	☐	20
Preparation of rounds is…	☐	☐	☐	☐	☐	☐	21
	1	2	3	4	5	6	

Personal information

When did you complete this questionnaire?

Date ☐☐ ☐☐ ☐☐☐☐ 22

**Your age**	**Your gender**
☐☐ years 23	☐ Male [1] ☐ Female [2] 24

Which formal training or degree have you completed? *(Check all that apply)* 25

- ☐ 1 Elderly care nurse
- ☐ 2 Nurse
- ☐ 3 Children's nurse
- ☐ 4 Occupational therapist
- ☐ 5 Other, please specify:______________________ 6

How many years have you worked at your facility? 26

- ☐ 1 less than 2 years
- ☐ 1 2 to 5 years
- ☐ 2 6 to 10 years
- ☐ 3 11 to 15 years
- ☐ 4 more than 15 years

Do you hold an executive or supervisory position at your facility? 27

- ☐ 1 no
- ☐ 2 yes, middle management
- ☐ 3 yes, upper management

What shifts do you usually work? „Day shift" may include early morning or late shifts. 28

- ☐ 1 day shifts mostly or exclusively
- ☐ 2 overnight shifts mostly or exclusively
- ☐ 3 both day and overnight shifts

If you have any comments, questions or concerns, please provide them here: 29

____

____

____

____

Thank you for your support!
