# Supplementary material for: A complex health services intervention to improve medical care in long-term care homes: study protocol of the controlled coordinated medical care (CoCare) study
Source: BMC Health Serv Res. 2019 May 24;19:332. doi: 10.1186/s12913-019-4156-4 (PMC6534891; doi:10.1186/s12913-019-4156-4)
Supplement: Supplementary file 7 — Doctor questionnaire follow-up. (PDF 311 kb) [file 12913_2019_4156_MOESM7_ESM.pdf]

Project
Extended coordinated medical care in nursing homes
(CoCare)

Questionnaire for doctors
2nd survey (follow-up)

Intervention group

Section of Health Care Research and Rehabilitation Research

Instructions for completing the questionnaire

With the following questions, we'd like to know more about your work in the enrolled nursing home and how you experience the cooperation with nursing staff.

Please answer these questions one after another in the sequence they appear on the questionnaire. **Please don't skip any questions.**

- ✓ You will answer most of the questions by checking a box. Please **check only one box per question.**
- ✓ **Please choose one of the options provided**! For some questions, you may need to fill in dates, numbers or text.
- ✓ If you feel like a question doesn't apply to you, **please check a box regardless**. Most of the time, one of the options provided will be an appropriate answer, e.g. "don't know" or "doesn't apply".
- ✓ A few questions will be very similar to one another, since this questionnaire consists of several shorter questionnaires. **Please answer all the questions regardless of repetition.**
- ✓ In case you need to correct an answer (i.e. you accidently checked the wrong box), please do so by filling in the falsely checked box completely. You may now check the correct box:

**Example question:**	**strongly agree**	**agree**	**agree some-what**	**disagree some-what**	**dis-agree**	**strongly dis-agree**
I actively participated in the discussion.	☐	■	☒	☐	☐	☐

Thank you for taking the time to participate in this survey!

Assessment of cooperation

The following questions concern the collaboration of all enrolled nursing staff and general practitioners/specialists involved in caring for nursing home residents. Please indicate to which extent the following statements apply to you.

Please refer your answers to the time **since CoCare has been implemented** at your facility.

Please choose only *one* option per item.

	strongly disagree	disagree	agree	strongly agree	don't know	
	☐	☐	☐	☐	☐	1
	☐	☐	☐	☐	☐	2
	☐	☐	☐	☐	☐	3
	☐	☐	☐	☐	☐	4
	☐	☐	☐	☐	☐	5
	☐	☐	☐	☐	☐	6
	1	2	3	4	5	

Questions 1 through 6 are adapted from the Internal Participation Scale (IPS):

Körner, M., & Wirtz, M. A. (2013). Development and psychometric properties of a scale for measuring internal participation from a patient and health care professional perspective. *BMC health services research*, *13*(1), 374.

The following questions concern the collaboration with nursing staff at the facility you provide medical care for. Please choose one of the response options and indicate to which extent the chosen option applies to you.

Please refer your answers to the time **since CoCare has been implemented** at your facility.

**very good**					**very poor**	7
☐	☐	☐	☐	☐	☐	
**1**	**2**	**3**	**4**	**5**	**6**	
1	2	3	4	5	6	

**very good**					**very poor**	**don't know**	8
☐	[illegible]	[illegible]	[illegible]	[illegible]	[illegible]	☐	
**1**	[illegible]	[illegible]	[illegible]	[illegible]	[illegible]	**7**	
1	[illegible]	[illegible]	[illegible]	[illegible]	[illegible]	7	

Questions 7 through 12 are adapted from a questionnaire assessing work satisfaction for doctors (Fragebogen zur Arbeitssituation von Ärzten; FAÄ):

Fischbeck, S., & Laubach, W. (2005). Arbeitssituation und Mitarbeiterzufriedenheit in einem Universitätsklinikum: Entwicklung von Messinstrumenten für ärztliches und pflegerisches Personal. *PPmP-Psychotherapie· Psychosomatik· Medizinische Psychologie*, *55*(06), 305-314.

**very much**					**very little**	9
☐	☐	☐	☐	☐	☐	
**1**	**2**	**3**	**4**	**5**	**6**	
1	2	3	4	5	6	

**very much**					**very little**	10
☐	☐	☐	☐	☐	☐	
**1**	**2**	**3**	**4**	**5**	**6**	
1	2	3	4	5	6	

very much					very little	11
☐	☐	☐	☐	☐	☐	
**1**	**2**	**3**	**4**	**5**	**6**	
1	2	3	4	5	6	

absolutely sufficient					absolutely insufficient	12
☐	☐	☐	☐	☐	☐	
**1**	**2**	**3**	**4**	**5**	**6**	
1	2	3	4	5	6	

Project satisfaction

In the past few months, CoCare has introduced a number of measures to improve medical care at nursing home facilities. Please assess how useful those measures were to you and your nursing home with these following questions.

How useful was the preparation of rounds by nursing staff or nursing home coordinators?

- ☐ 1 Very useful 13
- ☐ 2 Useful
- ☐ 3 Hardly useful
- ☐ 4 Not useful at all
- ☐ 5 To my knowledge, rounds weren’t prepared by nursing staff.

How useful was the joint documentation in CoCare-Cockpit (patients’ shared medical records)?

- ☐ 1 Very useful 14
- ☐ 2 Useful
- ☐ 3 Hardly useful
- ☐ 4 Not useful at all
- ☐ 5 To my knowledge, there was no documentation in CoCare-Cockpit.

How useful was having (a) fixed point/s of contact among nursing staff at the facility?

- ☐ 1 Very useful 15
- ☐ 2 Useful
- ☐ 3 Hardly useful
- ☐ 4 Not useful at all
- ☐ 5 To my knowledge, there was no fixed point of contact.

How useful were case conferences with doctors and assigned nursing staff?

- ☐ 1 Very useful 16
- ☐ 2 Useful
- ☐ 3 Hardly useful
- ☐ 4 Not useful at all
- ☐ 5 To my knowledge, no case conferences were held with doctors and assigned nursing staff, even though they might have been beneficial.
- ☐ 6 Not applicable

How useful were CoCare-recommended standard courses of treatment?

- ☐ 1 Very useful 17
- ☐ 2 Useful
- ☐ 3 Hardly useful
- ☐ 4 Not useful at all
- ☐ 5 I don’t know any CoCare-recommended standard courses of treatment.

How useful was the coordinated catheter management (insertion or change of suprapubic catheters at the facility, availability of portable ultrasound machines upon doctor’s request)?

- ☐ 1 Very useful 18
- ☐ 2 Useful
- ☐ 3 Hardly useful
- ☐ 4 Not useful at all
- ☐ 5 To my knowledge, there was no coordinated catheter management.

How useful was the coordinated medication management (regularly scheduled medication checks, interdisciplinary medication plans)?

- ☐ 1 Very useful 19
- ☐ 2 Useful
- ☐ 3 Hardly useful
- ☐ 4 Not useful at all
- ☐ 5 To my knowledge, no coordinated medication management was implemented at the facility.

You are enrolled in project CoCare as a… 20

☐ General practitioner	☐ Specialist
→as a general practitioner, please continue to answer the following questions. 1	→ as a specialist, please skip the next few questions and continue with the last question on **page 9.** 2

Questions for general practitioners only

How useful was the consolidation of general practitioners into GP-teams? 21

- ☐ 1 Very useful
- ☐ 2 Useful
- ☐ 3 Hardly useful
- ☐ 4 Not useful at all
- ☐ 5 To my knowledge, there were no GP-teams.

How useful did you find the quarterly meetings with nursing home coordinators and the GP-team? 22

- ☐ 1 Very useful
- ☐ 2 Useful
- ☐ 3 Hardly useful
- ☐ 4 Not useful at all
- ☐ 5 To my knowledge, no quarterly meetings were held.

Questions for general practitioners only

How useful did you find the weekly rounds with nursing staff and other general practitioners? 23

- ☐ 1 Very useful
- ☐ 2 Useful
- ☐ 3 Hardly useful
- ☐ 4 Not useful at all
- ☐ 5 To my knowledge, there were no weekly rounds with nursing staff and general practitioners.

How useful were the CoCare training courses for nursing staff and doctors? 24

- ☐ 1 Very useful
- ☐ 2 Useful
- ☐ 3 Hardly useful
- ☐ 4 Not useful at all
- ☐ 5 To my knowledge, no training courses were offered.

Questions for specialists only

How useful were the joint, quarterly rounds with specialists and nursing staff? 25

- ☐ 1 Very useful
- ☐ 2 Useful
- ☐ 3 Hardly useful
- ☐ 4 Not useful at all
- ☐ 5 To my knowledge, there were no quarterly rounds with specialists and nursing staff.

Starting here: Questions for both **general practitioners and specialists**

Changes in quality of care

The following questions ask you to assess the changes in the quality of care provided to nursing home residents that occurred due to the measures implemented by CoCare.

Please choose only *one* option per item.

	strongly disagree	disagree	agree	strongly agree	don't know	
Cooperation between doctors and nursing staff concerning patient care has been improved by CoCare.	☐	☐	☐	☐	☐	26
Organisation of rounds has been improved by CoCare.	☐	☐	☐	☐	☐	27
Documentation of rounds has been improved by CoCare.	☐	☐	☐	☐	☐	28
The implementation of doctor's orders has been improved by CoCare.	☐	☐	☐	☐	☐	29
Nursing staff's preparation of rounds has been improved by CoCare.	☐	☐	☐	☐	☐	30
	1	2	3	4	5	

Personal information

When did you complete this questionnaire?

Date ☐☐ ☐☐ ☐☐☐☐ 31

**Your age**	**Your gender**	
☐☐ years 32	☐ Male 1	☐ Female 2 33

You are enrolled in CoCare as…

☐ General practitioner 1

☐ Specialist 2 34

If you have any comments, questions or concerns, please provide them here: 35

____

____

____

____

Thank you for your support!
